# Prediction Model for Familial Aggregated HBV‐Associated Hepatocellular Carcinoma Based on Serum Biomarkers

**DOI:** 10.1002/cnr2.70253

**Published:** 2025-06-23

**Authors:** Linmei Zhong, Guole Nie, Qiaoping Wu, Honglong Zhang, Haiping Wang, Jun Yan

**Affiliations:** ^1^ Postgraduate Training Base Alliance, Wenzhou Medical University Wenzhou Zhejiang People's Republic of China; ^2^ Department of Colorectal Hernia Surgery Binzhou Medical University Hospital Binzhou Shandong People's Republic of China; ^3^ Department of Pediatrics The First Affiliated Hospital of Fujian Medical University Fuzhou Fujian People's Republic of China; ^4^ The First School of Clinical Medicine, Lanzhou University Lanzhou Gansu People's Republic of China; ^5^ Department of General Surgery The First Hospital of Lanzhou University Lanzhou Gansu People's Republic of China; ^6^ Key Laboratory of Biotherapy and Regenerative Medicine of Gansu Province Lanzhou Gansu People's Republic of China

**Keywords:** familial aggregated HBV, hepatocellular carcinoma, machine learning, risk prediction

## Abstract

**Background:**

Accurate assessment of the risk of familial aggregated hepatitis B virus (HBV)‐associated hepatocellular carcinoma (HCC) and regular surveillance for these patients at high risk may be valuable to reduce the occurrence and improve the prognosis of HCC.

**Aim:**

This study aimed to develop a simple and reliable prediction model for the risk of HCC in these patients.

**Methods and Results:**

This study analyzed clinical laboratory results from a database of 1285 patients with familial aggregated HBV who attended the First Hospital of Lanzhou University from January 2010 to December 2019. Univariate and multivariate logistic regression (LR) analysis showed that hemoglobin (Hb), neutrophil percentage (NP), total protein (TP), glutamyl transpeptidase (GGT), alglucosidase alfa (AFU), aspartate aminotransferase (AST) to Alanine aminotransferase (ALT) ratio (AAR), and alpha‐fetoprotein (AFP) were identified to be independent risk factors for HBV‐associated HCC. Prediction models were developed using a multivariate LR model, classification and regression tree, Native Bayes, Bagged tree, AdaBoost, and random forest. We used a multivariate LR model as a benchmark for performance assessment (AUC = 0.737). The results showed that the Native Bayes model had an AUC of 0.749, which was better than that of the other models.

**Conclusion:**

Finally, the Native Bayes model demonstrated better predictive performance for HCC, which helped in the clinical decision‐making and identification of HCC high‐risk groups.

## 1 Introduction

Globally, liver cancer is one of the most common tumors and has the third highest mortality rate among causes of cancer deaths, with approximately 906 000 new cases and 830 000 deaths worldwide in 2020 [1]. Hepatocellular carcinoma (HCC) is the main type of primary liver cancer, accounting for approximately 75%–85% of liver cancer cases [1]. The early symptoms of HCC are unapparent, and most cases of patients with HCC are clinically detected in the middle and late stages, with a recurrence rate of approximately 70% at 5 years after hepatectomy [2]. Therefore, early diagnosis of HCC is an important measure to improve the outcome of HCC.

The diagnosis of HCC currently relies on imaging and serological indices. Additionally, ultrasound with or without serum fetoprotein screening is a common screening tool for HCC. The sensitivity (Se) and specificity (Sp) of ultrasound have been reported to be 45%–78% and 80%–97%, respectively, while those of alpha‐fetoprotein (AFP) are 41%–60% and 70%–93%, respectively. The Se and Sp of ultrasound with AFP are 63%–85% and 80%–87%, respectively [3]. Ultrasound results are inevitably influenced by the operators and patients. Moreover, according to international guidelines, HCC surveillance is cost‐effective in patients with cirrhosis only if the annual incidence of HCC exceeds 1.5%–2% [4, 5], and there is insufficient evidence that HCC screening is beneficial in a large population. Therefore, the selection of high‐risk groups and the exploration of new screening programs that can be applied and promoted on a large scale are the focus of HCC prevention.

A significant contributing factor to HCC is chronic hepatitis B virus (HBV) infection, with over 50% of patients with HCC worldwide infected with it [6]. Among these, 75%–90% of HCC cases in HBV endemic areas are directly caused by HBV. Notably, for patients with HBV, effective means of HCC surveillance can reduce the occurrence of adverse outcomes. According to specific suggestions, early chronic HBV infection caused by the intrafamilial transmission of HBV may be associated with the familial aggregation of HCC [7]. Therefore, this research was conducted to investigate the correlation between changes in relevant clinical indicators and hepatocarcinogenesis in patients with familial aggregated HBV and establish a diagnostic model to decrease the cost of surveillance and guide clinical practice.

Machine learning methods, such as classification and regression trees (CART), AdaBoost, bagged tree (BT), Naive Bayes (NB), and random forest (RF), are widely used in clinical prediction and prognosis decisions. Naive Bayes classifiers, derived from Bayes' theorem, are simple and efficient and can be used to predict the class of new events. Unlike traditional or non‐Naive Bayesian classifiers, Naive Bayes uses a computationally easier learning process while still maintaining an excellent classification performance [8]. It is shown that naive Bayesian classifiers perform better than classifiers that take dependencies into account, such as AdaBoost, random forests, and support vector machines [9]. Its performance is equivalent to more complex classifiers such as neuro‐fuzzy, support vector machines, and artificial neural networks [10].

Previous studies have identified new diagnostic markers for HBV‐associated hepatocellular carcinoma, such as AFP‐L3 and PIVKA‐II, which have been found to have high specificity but are not routinely used as clinical tests [11, 12]. Whether routine blood and biochemical tests can be used to screen serum biomarkers for the diagnosis of HBV‐associated hepatocellular carcinoma as routine tests has rarely been reported. Therefore, this study will investigate the serum biomarkers for the diagnosis of HBV‐associated hepatocellular carcinoma mainly based on routine blood and biochemical tests. In this study, we investigated the risk factors associated with the development of familial aggregated HBV‐related HCC based on the clinical records of patients with familial aggregated HBV diagnosed in the First Hospital of Lanzhou University during the past 10 years. Finally, we established and validated the predictive performance of different diagnostic models.

## 2 Methods

### 2.1 Study Design and Population

Patients with a positive family history of hepatitis B who attended the First Hospital of Lanzhou University from January 2010 to December 2019 were selected for this study. The inclusion criteria were as follows: (1) diagnosis of HBV infection if the serological test was positive for HBV surface antigen (HBsAg); (2) presence of a pattern of familial aggregated HBV (family history with at least 2 cases of HBsAg‐positive blood relatives); (3) complete clinical history. The following were the exclusion criteria: combined hepatitis C virus (HCV), hepatitis A virus (HAV), hepatitis E virus (HEV), human immune deficiency virus (HIV) infection, autoimmune liver disease, metastatic liver cancer, genetic metabolic liver disease, alcoholic liver disease, schistosomiasis liver disease, and liver disease of unknown etiology. Subsequently, patients in the cohort were randomly divided into training and testing sets in the ratio of 7:3 (Figure S1). The training set was used for model construction, while the testing set was used for model validation.

### 2.2 Data Collection

All epidemiological and clinical data, including baseline data, tumor markers, biochemical indices, liver function indices, and imaging indices of the patients, were collected primarily by retrieving case data of the participants and telephone follow‐up. Specifically, the data collected included gender, age, presence of HCC, lymphocyte to monocyte ratio (LMR), hemoglobin (Hb), neutrophil percentage (NP), high‐density lipoprotein cholesterol (HDL‐C), prothrombin time (PT), blood glucose (GLU), glutamyl transpeptidase (GGT), α‐2‐microglobulin, alanine aminotransferase (ALT), aspartate aminotransferase (AST), and AST to ALT ratio (AAR), carcinoembryonic antigen (CEA), alpha‐fetoprotein (AFP), hyaluronic acid (HA), liver cirrhosis, portal hypertension and ascites, red blood cells, white blood cells, platelets, absolute neutrophil count, total cholesterol (CHOL), total protein (TP), alkaline phosphatase (ALP), triglycerides (TG), prothrombin activity (PTA), alglucosidase alfa (AFU), α‐hydroxybutyrate dehydrogenase (HBD), albumin (ALB), globulin (GLB), lactate dehydrogenase (LDH), fibrinogen (FIB), serum ferritin (SF), alpha 1‐microglobulin, carbohydrate antigen 19‐9 (CA19‐9), laminin (LN), HBV deoxyribonucleic acid (DNA) measured by polymerase chain reaction (PCR), and serum conjugated bile acid (SCG). For each participant, clinical information was collected prior to the administration of therapy to avoid influencing the final results.

Firstly, univariate LR was used to analyze whether the above 39 indicators were associated with HBV‐related HCC. Subsequently, variables with *p* < 0.05 were included in multivariate LR, which revealed that seven indicators, including Hb, NP, TP, GGT, AFU, AAR, and AFP, are independent risk factors for HBV‐associated HCC.

### 2.3 Statistical Analysis

All statistical analyses in this research were performed using the R software package (Version 4.0.5). Univariate and multivariate LR analyses were used to screen risk factors associated with HBV‐related HCC. Next, confusion matrixes were used to calculate important parameters such as the Se and Sp of each model. The sensitivity of a predictive model is the ability of the model to correctly identify positive samples, that is, the proportion of all samples that the model correctly predicts as positive out of all samples that are actually positive. The higher the sensitivity, the better the model is at identifying positive samples. Specificity is the ability of the model to correctly identify negative samples, that is, the proportion of all samples that are actually negative that the model correctly predicts as negative. The higher the specificity, the better the model is at identifying negative samples. *p* < 0.05 indicated positive statistical significance.

**FIGURE 1:**
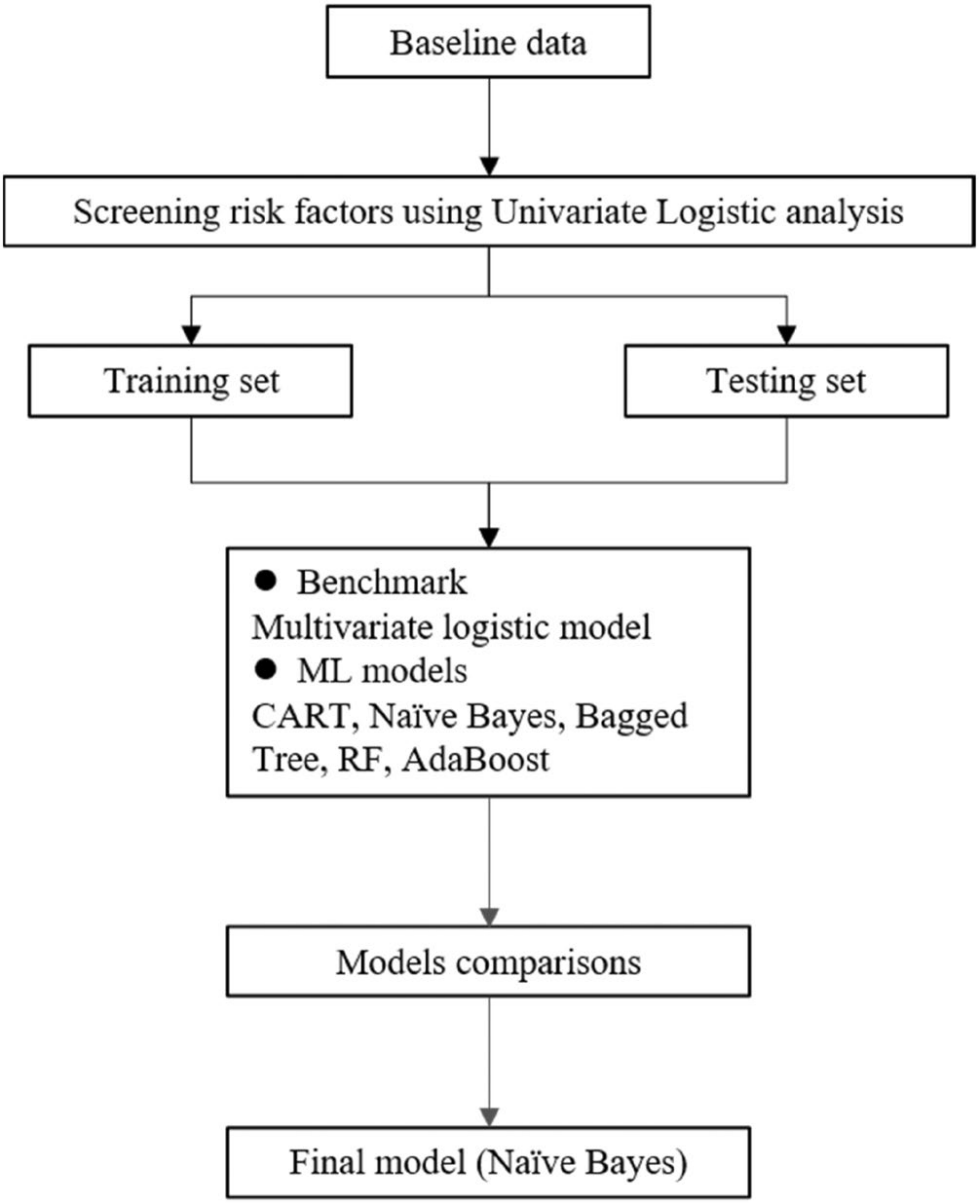
Flow chart of this study and the analytical flow of predictive model construction. ML, machine learning; CART, classification and regression trees; RF, random forest.

Furthermore, the receiver operating characteristics (ROC) curve was used to evaluate the discriminative ability of the various models. The model performs better when the area under the curve (AUC) is closer to 1, and more than 0.5 often implies that the model has a reference value [13]. The predictive performance of the different models was further verified using the Hosmer–Lemeshow goodness‐of‐fit test, and *p* < 0.05 indicated a statistically significant difference. A total of six models were constructed, including multivariate LR analysis, classification and regression trees (CART), AdaBoost, BT, NB, and random forest (RF). For the construction process of the specific model, we refer to the previous research literature [14]. The multivariate LR analysis was used as the benchmark for model performance evaluation to determine the best model, and we compared the prediction performance and related indexes among different models. Additionally, we set 100 trees in the RF and AdaBoost algorithms, and the rest of the parameters were default values. Figure 1 shows the analysis flow of this study.

## 3 Results

### 3.1 Baseline Characteristics of HBV‐Related Liver Cancer and Associated Risk Factors

Finally, 1285 patients, including 265 patients with HCC, were included in the database. Furthermore, all patients were randomly categorized into two groups as follows: the training group (*n* = 899) and the testing group (*n* = 386). The findings in this study showed a mean age of 52 years, with 378 women and 907 men (Table 1).

**TABLE 1:** Clinical baseline characteristics of patients with familial aggregation of HBV patients.

Characteristics	Non‐liver cancer (*n* = 1020)	Liver cancer (*n* = 265)	*p*
Male (%)	701 (68.7)	206 (77.7)	0.015
Age (mean ± SD)	51.70 ± 11.28	50.35 ± 10.91	0.131
Hb (mean ± SD)	125.71 ± 30.88	135.24 ± 29.00	< 0.001
NP (mean ± SD)	61.58 ± 13.15	66.35 ± 11.00	< 0.001
TP (mean ± SD)	65.52 ± 13.07	67.61 ± 13.63	0.022
GGT (mean ± SD)	74.06 ± 93.57	168.89 ± 185.56	< 0.001
AFU (mean ± SD)	23.41 ± 9.32	30.02 ± 16.05	< 0.001
AAR (mean ± SD)	1.25 ± 0.68	1.83 ± 2.11	< 0.001
AFP (mean ± SD)	87.28 ± 197.08	604.12 ± 3141.99	< 0.001

Abbreviations: AAR, aspartate aminotransferase (AST) to alanine aminotransferase (ALT) ratio; AFP, alpha‐fetoprotein; AFU, alglucosidase alfa; GGT, glutamyl transpeptidase; Hb, hemoglobin; NP, neutrophil percentage; SD, standard deviation; TP, total protein.

### 3.2 Cut‐Off Value of the Serum Biomarkers

**FIGURE 2:**
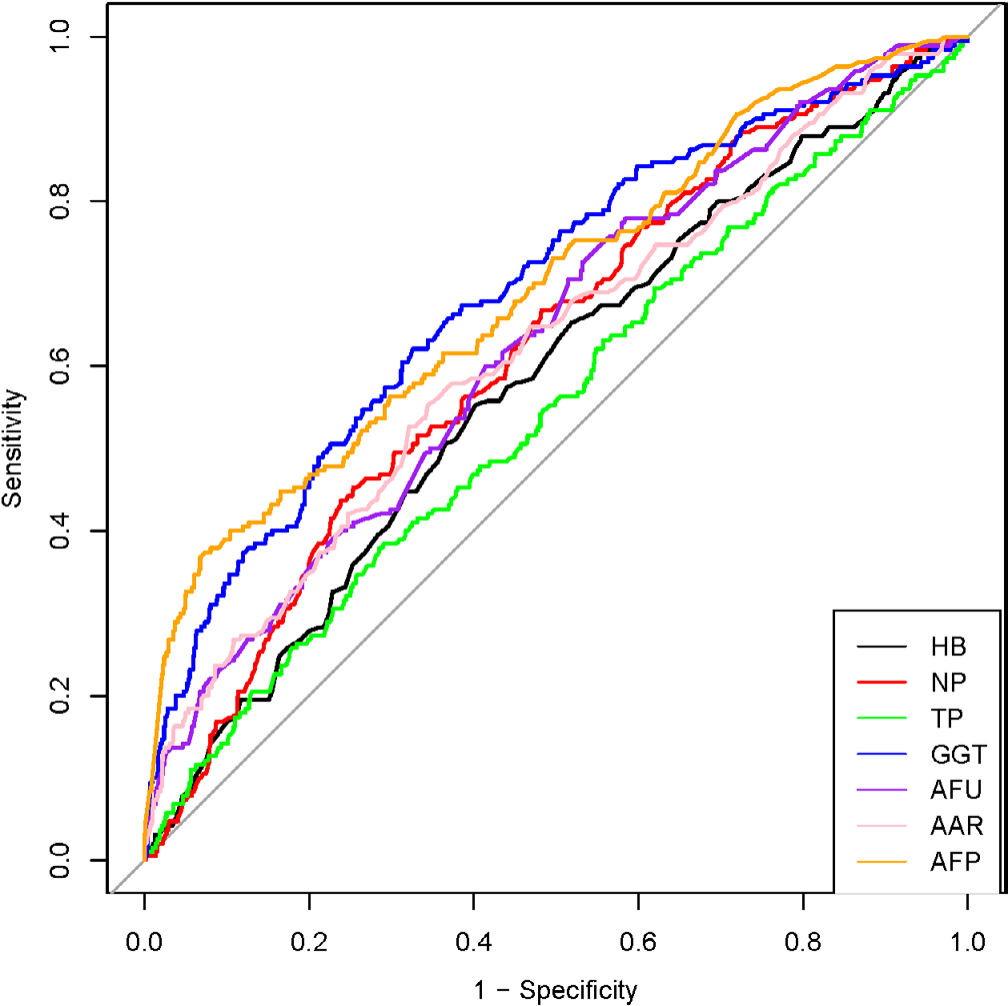
ROCs of different characteristics. The ROCs of each of the seven independent variables for the HCC prediction. The optimal cut‐off values for each were selected based on their respective ROCs. HB, hemoglobin; NP, neutrophil percentage; TP, total protein; GGT, glutamyl transpeptidase; AFU, alglucosidase alfa; AAR, aspartate aminotransferase (AST) to alanine aminotransferase (ALT) ratio; AFP, alpha‐fetoprotein.

The cut‐off values for each characteristic were Hb ≥ 136.5 (95% confidence interval (CI): 0.5359–0.6272), NP ≥ 69.85 (95% CI: 0.5776–0.6655), TP ≥ 72.67 (95% CI: 0.5015–0.5955), GGT ≥ 68.55 (95% CI: 0.6478–0.7354), AFU ≥ 20.405 (95% CI: 0.5858–0.6743), AAR ≥ 1.325 (95% CI: 0.5747–0.6671), and AFP ≥ 380.265 (95% CI: 0.6482–0.7366). The ROC curves evaluated the predictive power of variables and determined the cutoff values of each indicator based on the seven variables (Figure 2).

### 3.3 The Results of Univariate and Multivariate Logistic Analysis

Table 2 demonstrates the baseline information for the training and test groups. The results of univariate LR analysis showed that 39 indicators were associated with HCC. The results of the univariate analysis were combined using clinical expertise and literature, and we ultimately screened seven important predictive variables, which include Hb, NP, TP, GGT, AFU, AAR, and AFP. Moreover, the results of the univariate analysis showed that Hb, NP, TP, GGT, AFU, AAR, and AFP were all risk factors for the development of HBV‐associated HCC, and the multivariate logistic results showed that all seven indicators were independent risk factors for the development of HBV‐associated HCC (Table 3). We present a forest plot of the relevant variables in the model (Figure S2).

**TABLE 2:** Clinical baseline characteristics of the training and testing sets.

Characteristics	Training set (*n* = 899)	Testing set (*n* = 386)	*χ* ^2^	*p*
Liver cancer [*n* (%)]	190 (21.13)	75 (19.43)	0.042	0.489
Male [*n* (%)]	642 (71.41)	265 (68.83)	0.056	0.352
Age (mean ± SD)	52 ± 13.00	52 ± 11.25	0.889	0.011
Hb < 136.5 g/L [*n* (%)]	390 (43.38)	169 (43.78)	0.018	0.894
NP% ≥ 69.85% [*n* (%)]	266 (29.59)	115 (29.79)	0.005	0.941
TP ≥ 72.67 g/L [*n* (%)]	274 (30.48)	114 (29.53)	0.114	0.735
GGT ≥ 68.55 U/L [*n* (%)]	349 (38.82)	140 (36.27)	0.746	0.388
AFU ≥ 20.41 U/L [*n* (%)]	562 (62.51)	241 (62.44)	0.001	0.979
AAR ≥ 1.33 [*n* (%)]	349 (38.82)	159 (41.19)	0.635	0.426
AFP ≥ 380.26 ng/mL [*n* (%)]	118 (13.13)	40 (10.36)	1.912	0.167

Abbreviations: AAR, aspartate aminotransferase (AST) to alanine aminotransferase (ALT) ratio; AFP, alpha‐fetoprotein; AFU, alglucosidase alfa; GGT, glutamyl transpeptidase; Hb, hemoglobin; NP, neutrophil percentage; TP, total protein.

**TABLE 3:** Univariate and multivariate logistic results for the training group.

Characteristics	Univariate analysis			Multivariate analysis		
	OR	95% CI	*p*	OR	95% CI	*p*
AAR ≥ 1.33	2.354	1.701–3.259	0.000	2.311	1.683–3.174	0.000
AFP ≥ 380.26	8.033	5.301–12.172	0.000	5.287	3.567–7.837	0.000
AFU ≥ 20.41	2.511	1.728–3.649	0.000	1.505	1.038–2.184	0.031
GGT ≥ 68.55	3.391	2.432–4.729	0.000	1.728	1.234–2.421	0.001
Hb ≥ 136.5	1.838	1.331–2.538	0.000	1.593	1.135–2.238	0.007
NP ≥ 69.85	2.430	1.744–3.387	0.000	2.155	1.569–2.960	0.000
TP ≥ 72.67	1.531	1.095–2.142	0.013	1.497	1.083–2.069	0.014

Abbreviations: AAR, aspartate aminotransferase (AST) to alanine aminotransferase (ALT) ratio; AFP, alpha‐fetoprotein; AFU, alglucosidase alfa; GGT, glutamyl transpeptidase; Hb, hemoglobin; NP, neutrophil percentage; TP, total protein.

### 3.4 Construction and Evaluation of Predictive Models

**FIGURE 3:**
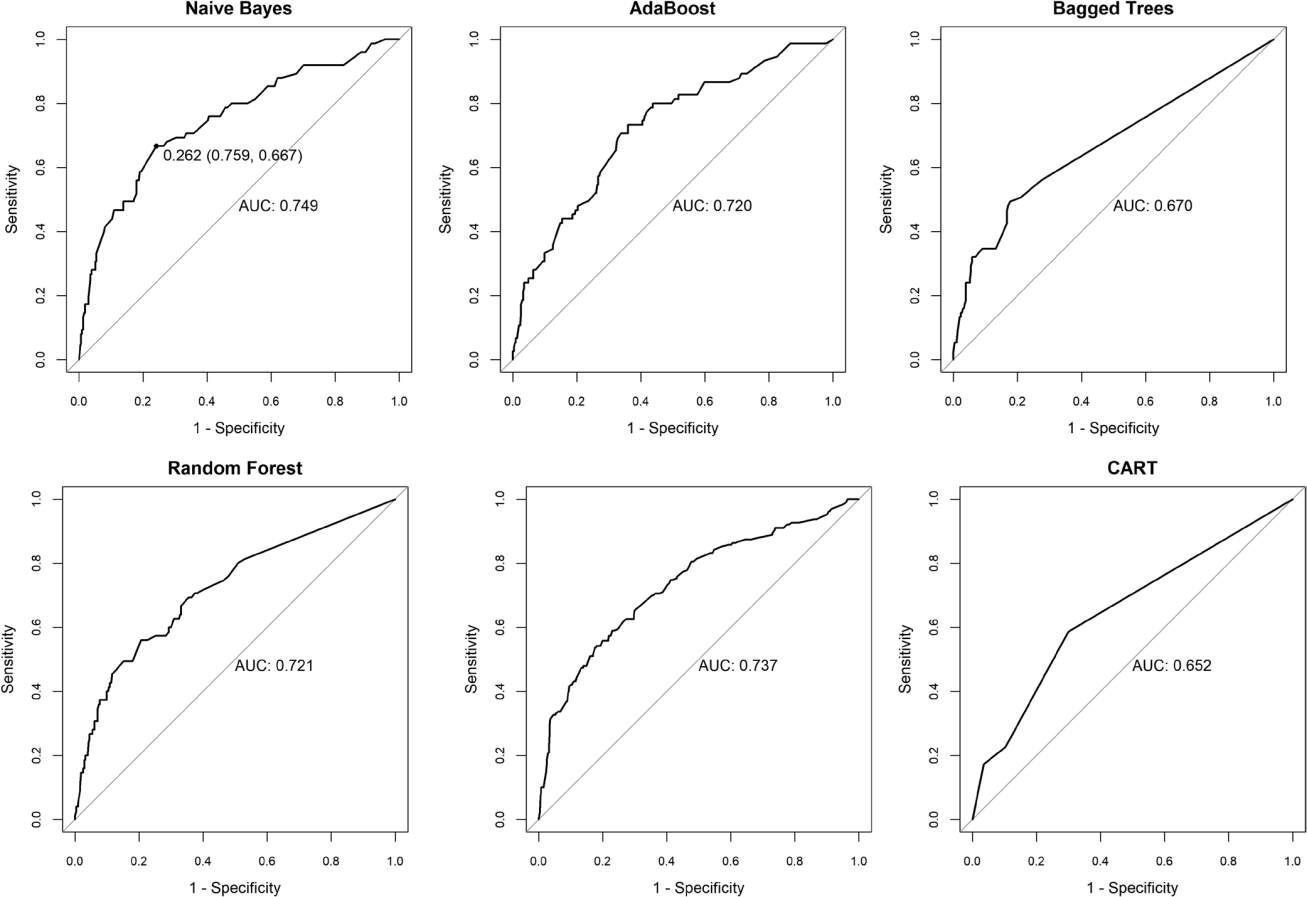
The ROCs of different prediction models. Predictive models were constructed using the seven independent variables screened, and the results of ROCs for six predictive models. CART, Classification and regression trees.

We randomly selected 70% of the total population as the training group for constructing the model and 30% as the testing group for validating the model. The results of the training and testing sets showed good consistency. Using multivariate LR, CART, NB, BT, AdaBoost, and RF methods, prediction models for familial aggregated HBV‐associated HCC were constructed in the training set based on the seven indicators screened above; furthermore, they were subsequently validated in the testing set. Additionally, we used the multivariate logistic model as the benchmark (AUC = 0.737) and compared the variation of the AUCs of the remaining five models (Table 4), and the ROCs of the different prediction models were plotted (Figure 3).

**TABLE 4:** AUC of the six prediction models.

Models	AUC	95% CI	AUC changes
LR	0.737	0.669–0.804	Benchmark
CART	0.652	0.588–0.717	−8.5%
NB	0.749	0.683–0.815	1.2%
BT	0.670	0.603–0.737	−6.7%
AdaBoost	0.720	0.655–0.785	−1.7%
RF	0.721	0.654–0.788	−1.6%

Abbreviations: BT, bagged tree; CART, classification and regression trees; LR, logistic regression; NB, Naive Bayes; RF, random forest.

Table 5 shows the Se, Sp, positive predictive value (PPV), negative predictive value (NPV), positive likelihood ratio (LR+), negative likelihood ratio (LR−), odds ratio (OR), and Youden Index of the six models. The results show that the plain Bayesian significantly improved over the benchmark (+1.2% changed in AUC) and outperformed the other models.

**TABLE 5:** Important evaluation parameters of the six prediction models.

Models	Se	Sp	PPV	NPV	LR+	LR−	OR	Youden index
LR	0.613	0.772	0.393	0.892	2.687	0.501	5.362	0.385
CART	0.587	0.701	0.321	0.876	1.962	0.590	3.327	0.288
NB	0.667	0.759	0.400	0.904	2.764	0.439	6.293	0.426
BT	0.493	0.823	0.402	0.871	2.790	0.616	4.532	0.316
AdaBoost	0.733	0.640	0.329	0.909	2.036	0.417	4.886	0.373
RF	0.560	0.794	0.396	0.882	2.721	0.554	4.912	0.354

Abbreviations: BT, bagged tree; CART, classification and regression trees; LR, logistic regression; LR−, negative likelihood ratio; LR+, positive likelihood ratio; NB, Naive Bayes; NPV, negative predictive value; OR, odds ratio; PPV, positive predictive value; RF, random forest; Se, sensitivity; Sp, specificity.

Based on the AUC comparison results of the above models in the testing group, we selected the plain Bayesian model with the best predictive efficacy and discriminative power (AUC = 0.749, 95% CI: 0.683–0.815) as the final risk prediction model. The Hosmer–Lemeshow test (*p* > 0.05) was used to assess the model's goodness of fit.

## 4 Discussion

It has been demonstrated that familial aggregated HCC and HBV have a synergistic effect and are associated with an over 70‐fold elevated HCC risk [15]. Familial aggregated HBV may be associated with polymorphisms in the vitamin D receptor (VDR) gene [16, 17]. 1,25(OH)2‐Vitamin D3 [1,25(OH)2D3] is the most active derivative of vitamin D, and when combined with VDR, the resulting product can inhibit HBV DNA replication in isolated liver tissue of HBV‐infected patients [18]. A study reported that when the viral genotypes of HBV‐infected patients from HBV‐clustering infection families (CIFs) were compared with HBV‐infected patients without a family history of infection, the prevalence of genotype C infection was significantly increased in the CIFs group, whereas genotype B was not present [19]. HBV genotype C is more likely to develop the end‐stage liver disease (liver cirrhosis and HCC) than genotype B [20].

Combining an individual risk‐stratification approach and tertiary prevention for HBV‐associated HCC would significantly reduce its global prevalence and improve the long‐term prognosis of HBV‐infected patients [21]. Hepatitis B vaccination and regular monitoring of HBV‐infected patients are the most effective means of controlling HBV‐associated HCC. Nevertheless, vaccination rates vary widely by region and population. In China, the number of unvaccinated infants remains at 10%, and many remain unprotected, especially those born before 2003 [22]. In addition, effective HBV vaccination has reduced the prevalence of HBV in children in the post‐vaccine era [23]. However, mother‐to‐child transmission of HBV has not been completely stopped. In a prospective study of 303 mothers and infants in Taiwan, all mothers were HBV carriers, and all infants received a complete immunization program; however, 3.3% of children still had chronic HBV infection [24]. In patients with familial aggregation of HBV, regular surveillance may be an essential tool to reduce the occurrence and improve the prognosis of HCC.

Currently, most scholars have defined high‐risk groups as residents of HBV endemic areas, chronic HBV carriers, patients with a family history of HCC, and patients with cirrhosis; they have also studied the risk prediction models for hepatocarcinogenesis in various high‐risk groups [25]. Ming‐Whei Yu et al. evaluated the risk of liver cancer in the relatives of chronic HBV (CHB) carriers with or without a family history of HCC for the first‐degree relatives of the Taiwanese cohort and showed that the cumulative risk of HCC at age 70 for HBV carriers with and without a family history was 235.6/1000 (95% CI: 95.3–375.9/1000) and 88.9/1000 (95% CI: 67.9–109.9/1000), respectively [26]. Risk estimation for HCC in chronic hepatitis B (REACH‐B) was conducted in Asian populations (mainly from Hong Kong, Taiwan, and Korea) of patients with CHB not receiving antiviral therapy and without cirrhosis, and it showed an area under the subject operating characteristic (AUROC) curve of 0.796 (95% CI: 0.775–0.816) for the 5‐year predicted risk of HCC occurrence, with associated risk factors being age, male gender, ALT, hepatitis Be antigen (HBeAg) positivity, and HBV DNA. In addition, there are prediction models for the risk of hepatocellular carcinogenesis in patients with CHB using antiviral drugs. For example, Yu J H et al. developed an HCC risk prediction model (age‐albumin‐sex‐liver cirrhosis‐HCC [AASL‐HCC] scoring system) for a cohort of 1242 patients with CHB treated with entecavir (ETV)/tenofovir disoproxil fumarate (TDF) in Korea, which had a C‐statistic of 0.802 (95% CI: 0.716–0.888) [27]. In contrast, this study developed an HBV‐related HCC risk prediction model based on patients with HBV who had a familial aggregated HBV background. The AUC of the NB model was 0.749, which indicated that the model had high diagnostic performance, optimized screening programs, and improved clinical decision‐making guides. Chinese patients account for over half of the global HCC burden, which is mainly associated with the prevalence of HBV [28]. Cost‐effective HCC surveillance measures for patients with CHB can effectively reduce the risk of HCC and save social resources. This study showed that Hb, NP, TP, GGT, AFU, AAR, and AFP are risk factors for the diagnosis of HCC. Notably, AFP and AFU are important tumor markers in the development of HCC. AFU is a widely available lysosomal enzyme, and its combination with AFP, which may be normal in 40% of patients with early‐stage HCC, may improve prediction model performance [29]. GGT, AAR, and TP are all sensitive indicators of liver function [30, 31]. Hb and NP may be related to the tumor microenvironment [32]. Notably, each factor can predict the occurrence of HCC to a degree, and clinical models can take advantage of each factor to provide more valuable predictions for clinicians.

HCC is the leading cause of cancer deaths worldwide. Serum biomarkers such as alpha‐fetoprotein (AFP), vitamin K deficiency‐II‐induced proteins, and AFP‐L3, AFP, des‐gamma‐carboxy prothrombin (DCP), and Des‐γ‐carboxyprothrombinogen (GALAD) scores have now been recommended for monitoring HCC [33, 34]. BAIAP2L2 is a novel prognostic biomarker related to the migration and invasion of HCC [35]. More and more new serum markers continue to be reported, and their emergence and application will be more beneficial for the future diagnosis of HCC.

In recent years, more machine learning models have been applied to clinical decision‐making, and it has shown better predictive performance in several fields. Therefore, to optimize the HVB‐related HCC risk prediction model, this study analyzed and compared various machine learning models with specific data and selected the best‐performing prediction model (NB) from the six prediction models (NB, RF, BT, CART, multivariate LR analysis, and Adaboost) as the final HBV‐related HCC risk prediction model applicable to HBV carriers with familial aggregation of HBV. NB was a classification method that was based on Bayesian theory, and it used the “attribute conditional independence assumption” to estimate the class prior probabilities based on the training set and the conditional probabilities for each attribute during the training process. Although “conditional independence” is unlikely to occur in most cases, NB has a remarkable competitive performance in the case of classification, which may be related to the local dependence distribution of attributes [36]. Additionally, NB allows incremental learning, which facilitates data analysis for subsequent studies and further optimization and improvement of the prediction.

There were some limitations of this study. First, this study is a single center retrospective research, and there was some bias in the population selection. The population in this study was mainly focused on Northwest China and was Chinese; therefore, variables such as ethnicity, socioeconomic status, and lifestyle have potential effects on the risk of HCC. These limitations, to some extent, also restrict the external application of the model. Therefore, follow‐up multicenter studies are necessary to explore these potential effects.

Currently, more and more studies are focusing on the application of predictive modeling in HBV‐associated hepatocellular carcinoma [37, 38, 39, 40]. Machine learning sheds light on prognostication for early‐stage hepatocellular carcinoma [41]. It was found that long‐term antiviral treatment in patients with chronic hepatitis B led to a decrease in the performance of hepatocellular carcinoma prediction models, which is important for the construction of HBV‐associated HCC prediction models that should fully take into account the effects of antiviral treatment [42, 43, 44]. However, there are few reports on familial aggregation of HBV‐associated HCC.

In patients with familial aggregation of HBV, emphasis should be placed on the dynamics of Hb, NP, TP, GGT, AFU, AAR and AFP during patient follow‐up. This is a preliminary study, and additional clinical bedside indicators (e.g., HBV viral load, liver fibrosis stage) will be included in the follow‐up study to further improve the predictive performance of the model, and to establish a more stable predictive model through multicenter studies to guide clinical practice.

In addition, the data collected in this study may not have been sufficient to train the NB algorithm, and more data could yield better AUC results. All these problems can be solved by expanding the study population, reaching multi‐center cooperation, and increasing the sample size to validate further and optimize the model.

## Author Contributions

All authors contributed to the study conception and design. Material preparation and data collection were performed by Linmei Zhong and Qiaoping Wu. Statistical analyses were performed by Guole Nie, Haiping Wang, and Honglong Zhang. The first draft of the manuscript was written by Linmei Zhong and Guole Nie. Jun Yan supervised the entire process and reviewed the final version of the manuscript. All authors commented on previous versions of the manuscript. All authors read and approved the final manuscript.

## Ethics Statement

All patients provided written consent for storage of their information in the hospital database and for use of this information for research purposes. The study was approved by the ethics committee of the First Hospital of Lanzhou University before the start of the study (ethical code: LDYYYLL2022‐378).

## Conflicts of Interest

The authors declare no conflicts of interest.

## Supporting information


**Figure S1.** Patient selection and distribution flowchart. According to the inclusion and exclusion criteria, 1285 patients were finally included and randomly divided into training set (*n* = 899) and testing set (*n* = 386) in a ratio of 7:3. HIV, human immunodeficiency virus.

**Figure S2.** A forest plot of the relevant variables. Shows the estimates and confidence intervals for the seven characteristics. In this figure, the horizontal axis represents the effect size of the feature in the model and the vertical axis lists the different variables. Each diamond in the figure represents the point estimate and 95% confidence interval of the effect size for the corresponding variable. Hb, hemoglobin; NP, neutrophil percentage; TP, total protein; GGT, glutamyl transpeptidase; AFU, alglucosidase alfa; AAR, aspartate aminotransferase (AST) to Alanine aminotransferase (ALT) ratio; AFP, alpha‐fetoprotein. Intercept, denotes the intercept in the model; Sigma, refers to the standard errors of the model.

## Data Availability

The data that support the findings of this study are available from the corresponding author upon reasonable request.
