# Supplementary figures and images for: Prediction Model for Familial Aggregated HBV‐Associated Hepatocellular Carcinoma Based on Serum Biomarkers
Source: Cancer Rep (Hoboken). 2025 Jun 23;8(6):e70253. doi: 10.1002/cnr2.70253 (PMC12185788; doi:10.1002/cnr2.70253)

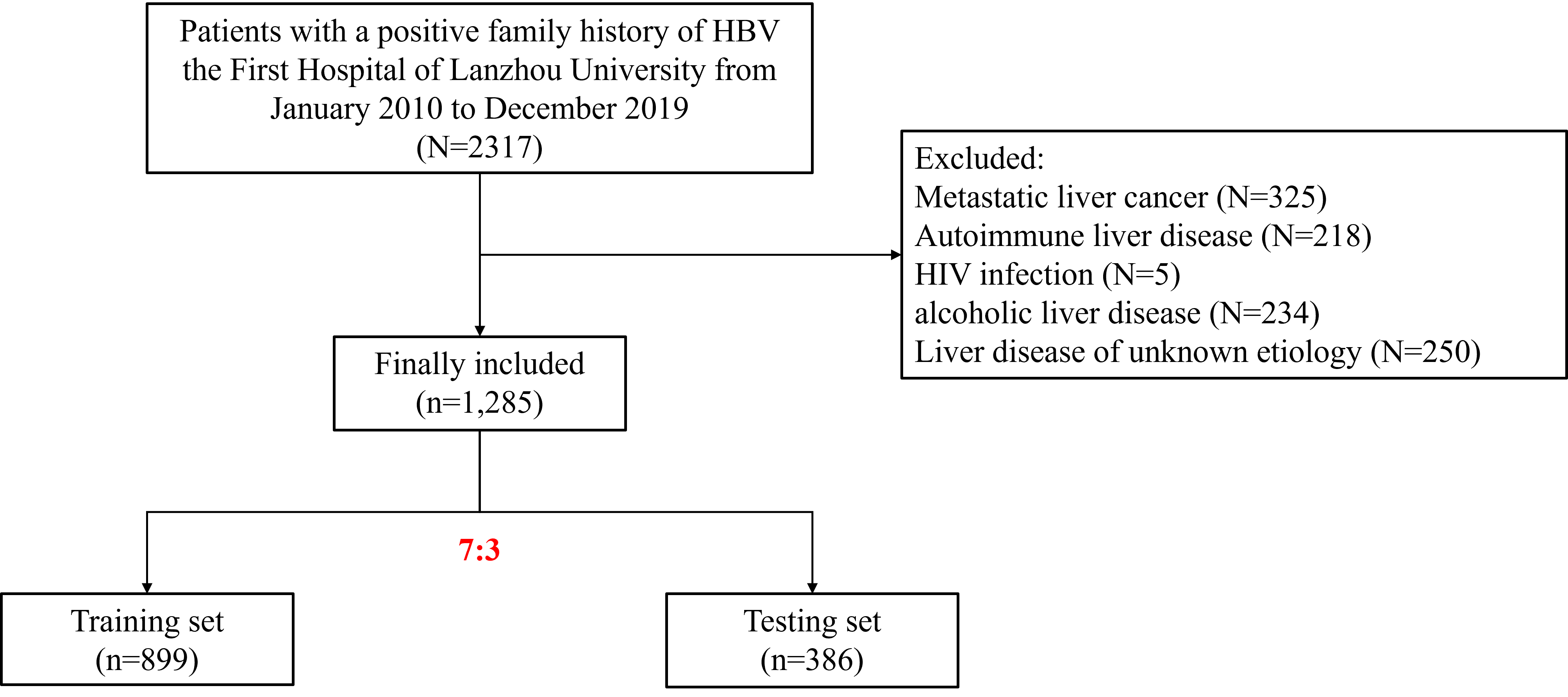

Supplement: Supplementary file 1 — Figure S1. Patient selection and distribution flowchart. According to the inclusion and exclusion criteria, 1285 patients were finally included and randomly divided into training set (n = 899) and testing set (n = 386) in a ratio of 7:3. HIV, human immunodeficiency virus. [file CNR2-8-e70253-s001.png]

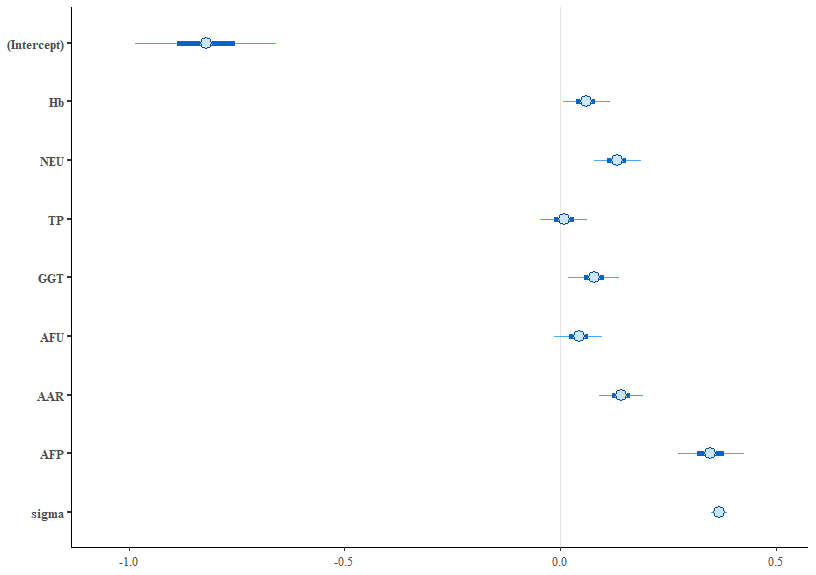

Supplement: Supplementary file 2 — Figure S2. A forest plot of the relevant variables. Shows the estimates and confidence intervals for the seven characteristics. In this figure, the horizontal axis represents the effect size of the feature in the model and the vertical axis lists the different variables. Each diamond in the figure represents the point estimate and 95% confidence interval of the effect size for the corresponding variable. Hb, hemoglobin; NP, neutrophil percentage; TP, total protein; GGT, glutamyl transpeptidase; AFU, alglucosidase alfa; AAR, aspartate aminotransferase (AST) to Alanine aminotransferase (ALT) ratio; AFP, alpha‐fetoprotein. Intercept, denotes the intercept in the model; Sigma, refers to the standard errors of the model. [file CNR2-8-e70253-s002.tiff]
